# Screening for Hepatocellular Carcinoma and Survival in Patients With Cirrhosis After Hepatitis C Virus Cure

**DOI:** 10.1001/jamanetworkopen.2024.20963

**Published:** 2024-07-10

**Authors:** Catherine Mezzacappa, Nicole J. Kim, Philip Vutien, David E. Kaplan, George N. Ioannou, Tamar H. Taddei

**Affiliations:** 1 Division of Digestive Diseases, Yale School of Medicine, New Haven, Connecticut; 2 Gastroenterology Section, VA Connecticut Healthcare System, West Haven, Connecticut; 3 Division of Gastroenterology, University of Washington, Seattle; 4 Gastroenterology Division, VA Puget Sound Health Care System, Seattle, Washington; 5 Division of Gastroenterology and Hepatology, University of Pennsylvania School of Medicine, Philadelphia; 6 Gastroenterology Section, Corporal Michael J. Crescenz Veterans Affairs Medical Center, Philadelphia, Pennsylvania

## Abstract

**Importance:**

The risk of hepatocellular carcinoma (HCC) declines over time after hepatitis C virus (HCV) cure by direct-acting antiviral (DAA) therapies. Liver society guidelines recommend continuing HCC screening for these patients, but data on screening outcomes are lacking.

**Objective:**

To evaluate the association of HCC screening after HCV cure with overall survival.

**Design, Setting, and Participants:**

This cohort study evaluated patients with HCV cirrhosis who achieved DAA-induced HCV cure in the Veterans Affairs health care system between January 2014 and December 2022. Data analysis occurred from October 2023 to January 2024.

**Exposures:**

The percentage of time spent up to date with recommended HCC screening was calculated by year of follow-up and during the 4 years preceding HCC diagnosis (the detectable asymptomatic phase).

**Main Outcomes and Measures:**

The primary outcome was overall survival after HCC diagnosis and was compared by percentage of time spent up to date with screening using Kaplan-Meier analyses and Cox proportional hazards regression. Early-stage HCC at diagnosis and curative treatment were secondary outcomes assessed using logistic regression.

**Results:**

A total of 16 902 individuals were included (median [IQR] age, 64.0 [60.5-67.4] years; 16 426 male [97.2%]), of whom 1622 developed HCC. The cumulative incidence of HCC declined from 2.4% (409 of 16 902 individuals) to 1.0% (27 of 2833 individuals) from year 1 to year 7 of follow-up. Being up to date with screening for at least 50% of time during the 4 years preceding HCC diagnosis was associated with improved overall survival (log-rank test of equality over strata *P* = .002). In multivariate analysis, each 10% increase in follow-up spent up to date with screening was associated with a 3.2% decrease in the hazard of death (hazard ratio, 0.97; 95% CI, 0.95-0.99). There was a statistically significant interaction between time since HCV cure and screening, with no association observed among those who received a diagnosis of HCC more than 5 years after HCV cure. Each 10% of time spent up to date with screening was associated with a 10.1% increased likelihood of diagnosis with early-stage HCC (95% CI, 6.3%-14.0%) and a 6.8% increased likelihood of curative treatment (95% CI, 2.8%-11.0%).

**Conclusions and Relevance:**

In this cohort study of persons with HCV-related cirrhosis who achieved HCV cure and subsequently developed HCC, remaining up to date with screening was associated with improved overall survival, supporting the screening of eligible individuals.

## Key Points

**Question:**

Is screening for hepatocellular carcinoma (HCC) in persons with hepatitis C virus (HCV)–associated cirrhosis after direct-acting antiviral (DAA) therapy associated with improved survival?

**Findings:**

In this cohort study of 16 902 people with HCV-associated cirrhosis who achieved HCV cure after treatment with DAA, rates of HCC declined gradually over 8 years of follow-up. Among 1622 persons who received a diagnosis of HCC, remaining up to date with recommended screening examinations was associated with improved overall survival after HCC diagnosis through 5 years after HCV cure.

**Meaning:**

These findings suggest that individuals with cirrhosis who have achieved HCV cure should receive recommended HCC screening.

## Teaser

This cohort study assesses whether screening for hepatocellular carcinoma (HCC) is associated with improved survival among individuals with hepatitis C virus (HCV)–associated cirrhosis who achieved HCV cure in the Veteran’s Affairs health system.

## Introduction

Hepatitis C virus (HCV) has been the most common cause of cirrhosis and hepatocellular carcinoma (HCC) in the US for decades.^1^ Direct-acting antivirals (DAAs) became available in 2014 and revolutionized HCV care, providing an efficacious and well-tolerated cure for HCV for millions of people in the US.^2,3^ In HCV-associated cirrhosis, early studies^4,5^ demonstrated a dramatic decline in HCC rates within 2 to 3 years following HCV treatment with DAAs. However, HCC incidence remained elevated for years afterward.^4,6,7,8,9^

Multiple observational studies have demonstrated associations of HCC screening, consisting of semiannual imaging and α-fetoprotein measurement, with improved detection of early-stage HCC, receipt of curative treatment, and overall survival.^10^ However, adherence to screening is poor, with approximately 1 in 4 eligible individuals receiving recommended screening.^11,12^ Individuals with HCV-associated cirrhosis who have achieved viral cure may be particularly susceptible to lapses in HCC screening due to treatment-induced stabilization of their liver disease.^13^ However, data on clinical HCC screening practices and outcomes in these patients are sparse.

Screening for HCC in cirrhosis is recommended by all global liver societies,^9,14,15^ but its benefits have been controversial.^16^ Of the 2.5 to 3.5 million individuals affected by HCV in the US, a majority were born between 1945 and 1965.^17^ This aging population is at risk for comorbid medical conditions that might attenuate the benefit of HCC screening.^18^ To balance its potential harms,^19,20^ it is important to establish the benefits of HCC screening in this population. The objectives of this study were to assess (1) whether HCC screening declines among persons with HCV-associated cirrhosis after HCV cure and (2) whether HCC screening remains associated with improved overall survival in older patients with cirrhosis who received a diagnosis of HCC in the Veterans Affairs (VA) health care system, the largest provider of HCV care in the US.^5^

## Methods

This is a retrospective cohort study of adults with HCV-associated cirrhosis who achieved DAA-induced viral cure in the VA health care system. This study received institutional review board approval from the VA Connecticut Healthcare System and followed the Strengthening the Reporting of Observational Studies in Epidemiology (STROBE) reporting guideline for cohort studies. This study was granted institutional review board exemption for informed consent because of the use of deidentified data, in accordance with 45 CFR §46. Development of this cohort has been described previously in detail.^21^ In brief, cirrhosis was identified using either 1 inpatient or 2 outpatient *International Classification of Diseases, Ninth Revision, Clinical Modification *(*ICD-9-CM*; 571.2 and 571.5) or *International Statistical Classification of Diseases and Related Health Problems, Tenth Revision *(*ICD-10*; K74.6x and K70.3x) diagnosis codes. Cause of cirrhosis was determined using a validated algorithm.^22^ Persons with cirrhosis attributed to HCV alone or HCV and alcohol use were considered for inclusion in the analytic cohort.

### Sample

All HCV-directed antiviral therapies were queried from pharmacy records in the VA Corporate Data Warehouse. Treatment with a DAA was identified using filled prescriptions for elbasvir-grazoprevir, glecaprevir-pibrentasvir, ledipasvir-sofosbuvir, sofosbuvir-velpatasvir, sofosbuvir, and daclatasvir. We identified 19 665 individuals with HCV-associated cirrhosis prescribed a DAA between 2014 and 2021 with a subsequent confirmatory negative HCV RNA polymerase chain reaction test. Individuals with advanced hepatic dysfunction, identified by Child-Turcotte-Pugh (CTP) class C cirrhosis, are unlikely to benefit from HCC treatment^23^ and are not recommended for HCC screening unless they are eligible for liver transplantation.^9^ We therefore excluded 1406 individuals with prevalent HCC, other metastatic cancer (captured using the cirrhosis-specific comorbidity [CirCom] score), or CTP class C cirrhosis before DAA treatment. We excluded individuals with less than 1 year of follow-up following initiation of DAA therapy (671 individuals). Patients who received a diagnosis of HCC within 1 year of DAA initiation (679 individuals) were also excluded to avoid capturing prevalent disease. An additional 7 individuals were missing follow-up status.

### Exposure: Percentage of Time Participants Were Up to Date With Screening During Eligible Follow-Up

Screening was measured as the percentage of time participants were up to date with screening during eligible follow-up. Individuals were considered eligible for HCC screening until development of HCC, other metastatic cancer, CTP class C cirrhosis, death, or end of follow-up. All abdominal ultrasonography and contrast-enhanced computed tomography and magnetic resonance imaging examinations obtained during eligible follow-up were identified. Individuals were considered up to date with screening for up to 180 days after a qualifying imaging study or until their next study if it occurred within less than 180 days.^24^ Total days up to date with screening was divided by screening-eligible follow-up days to define the percentage of time participants were up to date with screening overall and by year since HCV cure. To evaluate the association of HCC screening with survival after HCC diagnosis, the percentage of time participants were up to date with screening during the 4 years preceding HCC diagnosis (the detectable preclinical phase of HCC^25^) was calculated. Individuals who remained up to date with screening for at least 50% of each year during up to 4 years preceding HCC diagnosis were classified as consistently up to date with screening.

### Other Patient Characteristics

Patient demographics including age, sex, race, and ethnicity were ascertained from the medical record. Race and ethnicity categories included American Indian, Asian or Pacific Islander, Black, Hispanic, White, and other (ie, individuals who identified as any race or ethnicity not otherwise specified or individuals with unknown race and ethnicity). Hispanic ethnicity included any race, whereas non-Hispanic Black individuals and non-Hispanic White individuals were classified as Black and White, respectively. Race and ethnicity were included to ensure the diversity of the sample.

The CTP class is a measure of the degree of decompensation in liver cirrhosis. Class A corresponds to good hepatic function, class B to moderately impaired function, and class C to advanced hepatic dysfunction. This was assessed using a validated algorithm^26^ at baseline and for every 30-day window of follow-up. The CTP class at time of HCC diagnosis was identified where applicable.

Comorbid health conditions were assessed using the CirCom score, a validated scoring system that estimates mortality in persons with cirrhosis more accurately than the Charlson Comorbidity Index.^27^ Details of the CirCom score are included in eTable 1 in Supplement 1. The CirCom score at time of HCC diagnosis was identified where applicable.

### Outcomes

The primary outcomes of this study were incident HCC (measured in the total sample) and survival after diagnosis of HCC. The index date was the date of first DAA prescription that was followed by a negative HCV RNA polymerase chain reaction test 12 weeks after therapy completion. Patients were followed up for incident HCC and death beginning 1 year from the index date. Patients who did not develop HCC or die were censored at their last follow-up or on December 31, 2022, whichever came first. Incident HCC was defined using 1 inpatient or 2 outpatient *ICD-9-CM *and *International Statistical Classification of Diseases, Tenth Revision, Clinical Modification (ICD-10-CM) *primary or secondary diagnosis codes for malignant neoplasm of the liver or liver cell carcinoma (*ICD9-CM*, 155.0 and 155.2; *ICD10-CM*, C22.0) with no *ICD-9-CM* or *ICD-10-CM* codes for cholangiocarcinoma (*ICD-9-CM*, 155.1; *ICD-10-CM*, C22.1). These codes have a positive predictive value of 78.2% for confirmed HCC in VA data.^28^ All-cause mortality and date of death were ascertained using the VA Vital Status Master File.

Cancer stage at HCC diagnosis was defined using the American Joint Commission on Cancer (AJCC) staging manual, which classifies liver cancers by stage groupings from I (least advanced) to IV (most advanced).^29^ In patients with HCC, multifocal disease and microvascular invasion (characteristics of AJCC stage II disease) are poor prognostic markers.^30^ Therefore, stage at diagnosis was categorized as early (AJCC stage I) vs all other stages.

The first HCC-directed treatment received (defined as surgical resection, ablation, embolization, radiation, or systemic or supportive therapy) was classified using VA data tables as described previously (eTable 2 in Supplement 1).^28^ Resection and ablation are potentially curative therapies for HCC.

### Statistical Analysis

The analytic sample was described in total and stratified by percentage of time participants were up to date with screening during eligible follow-up (<50% vs ≥50% of eligible follow-up time). Medians with IQRs were calculated for age and compared using Wilcoxon rank-sum tests, and percentages were calculated for categorical variables and compared using χ^2^ tests.

The mean percentage of time participants were up to date with screening was calculated by year since HCV cure for up to a maximum of 8 years. Annual cumulative incidence of death, HCC, other metastatic cancer, and CTP class C cirrhosis were calculated for each year of follow-up.

Kaplan-Meier curves estimating survival after HCC diagnosis were calculated and stratified by percentage of time spent up to date with screening in the 4 years preceding HCC diagnosis (<50% vs ≥50%). Cox proportional hazards regression was used to evaluate overall survival after diagnosis of HCC and percentage of eligible follow-up time up to date with screening in the 4 years preceding HCC diagnosis. Time 0 was date of HCC diagnosis, and data were right-censored at loss to follow-up or maximum follow-up. These models were adjusted for age at HCC diagnosis, sex, race and ethnicity, cirrhosis due to HCV alone vs HCV and alcohol, number of years since HCV cure, CTP class at HCC diagnosis, CirCom score at HCC diagnosis, and smoking status. An interaction term between time since HCV cure and percentage of time up to date with screening was used to assess for statistical interaction. A secondary analysis was performed stratified by consistently vs inconsistently in screening. Logistic regression was used to assess the likelihood of early-stage HCC and likelihood of curative treatment receipt.

Sensitivity analyses stratified by age at HCC diagnosis (<70 vs ≥70 years and <75 vs ≥75 years) and evaluation screening during the 2 years preceding HCC diagnosis were performed. To account for lead-time bias, a further sensitivity analysis adjusted observed survival time for individuals up to date with screening for 40% or more of follow-up for the median estimated lead-time associated with annual screening (4.1 months).^31^

Comparisons were considered statistically significant at 2-tailed *P* < .05. All data analyses were performed using SAS Enterprise Guide version 8.3 (SAS Institute). Data analysis was conducted from October 2023 to January 2024.

## Results

### Characteristics of the Study Population

The analytic sample included 16 902 adults with HCV-associated cirrhosis (median [IQR] age, 64.0 [60.5-67.4] years; 16 426 male [97.2%]; 176 American Indian [1.0%]; 193 Asian [1.1%]; 5929 Black [35.1%]; 1258 Hispanic [7.4%]; 8354 White [49.4%]; and 992 other [5.9%]). At baseline, 14 609 (86.4%) had CTP class A cirrhosis and the remainder had CTP class B cirrhosis. One-third actively smoked tobacco (5573 individuals [33.0%]) or previously smoked tobacco (6072 individuals [35.9%]). Of the 1622 individuals who received a diagnosis of HCC (median [IQR] age, 66.3 [63.7-69.7] years), 856 (52.8%) had cirrhosis due to HCV alone, and CirCom scores at HCC diagnosis reflected a range of comorbid disease (757 individuals [46.6%] had a CirCom score of 3 + 0 or greater). Of the patients who received a diagnosis of HCC, 74 (4.6%) underwent surgical resection as their first HCC treatment. Years from HCV cure to HCC and first HCC treatment received differed between individuals who were up to date with screening for less than 50% and 50% or more of eligible follow-up during the 4 years preceding HCC diagnosis. Other characteristics were similar across these exposure groups (Table 1).

**Table 1.** Characteristics of Participants With HCV Cirrhosis Who Achieved Viral Cure and Participants Who Received a Diagnosis of HCC

Characteristic	Participants with HCV cirrhosis who achieved viral cure, No. (%)^a^				Subset who received diagnosis of HCC during follow-up, values at HCC diagnosis, No. (%)^b^			
	Total sample (N = 16 902)	Screening up to date <50% of follow-up time (n = 8223)	Screening up to date ≥50% of follow-up time (n = 8679)	*P* value^c^	Total sample (N = 1622)	Screening up to date <50% of 4 y preceding HCC (n = 519)	Screening up to date ≥50% of 4 y preceding HCC (n = 1103)	*P* value^d^
Age, median (IQR), y	64.0 (60.5-67.4)	63.5 (60.0-67.1)	64.4 (60.9-67.6)	<.001	66.3 (63.3-69.6)	66.6 (63.7-69.7)	66.3 (63.3-69.6)	.12
Sex								
Male	16 426 (97.2)	7969 (96.9)	8457 (97.4)	.04	1589 (98.0)	507 (97.7)	1082(98.1)	.59
Female	476 (2.8)	254 (3.1)	222 (2.6)		33 (2.0)	12 (2.3)	21 (1.9)	
Race and ethnicity								
American Indian	176 (1.0)	90 (1.1)	86 (1.0)	.40	20 (1.2)	6 (1.2)	14 (1.3)	.44
Asian or Pacific Islander	193 (1.1)	91 (1.1)	102 (1.2)		22 (1.4)	7 (1.4)	15 (1.4)	
Black	5929 (35.1)	2894 (35.2)	3035 (35.0)		457 (28.2)	142 (27.4)	315 (28.6)	
Hispanic	1258 (7.4)	586 (7.1)	672 (7.7)		132 (8.1)	46 (8.9)	86 (7.8)	
White	8354 (49.4)	4100 (49.9)	4254 (49.0)		881 (54.3)	292 (56.3)	589 (53.4)	
Other^e^	992 (5.9)	462 (5.6)	530 (6.1)		110 (6.8)	26 (5.0)	84 (7.6)	
Cause of cirrhosis								
HCV alone	9872 (58.4)	4662 (56.7)	5210 (60.0)	<.001	856 (52.8)	268 (51.6)	588 (53.3)	.53
HCV and alcohol	7030 (41.6)	3561(43.3)	3469 (40.0)		766 (47.2)	251 (48.4)	515 (46.7)	
Baseline Child-Turcotte-Pugh class								
A	14 609 (86.4)	7216 (87.7)	7393 (85.2)	<.001	1430 (88.2)	467 (90.0)	963 (87.3)	.12
B	2293 (13.6)	1007 (12.3)	1286 (14.8)		192 (11.8)	52 (10.0)	140 (12.7)	
Tobacco use								
Never	5068 (30.0)	2405 (29.3)	2663 (30.8)	.002	462 (28.4)	154 (29.8)	308 (28.2)	.44
Former	6072 (35.9)	2915 (35.5)	3157 (36.3)		607 (37.4)	183 (35.5)	424 (38.8)	
Current	5573 (33.0)	2823 (34.4)	2750 (31.9)		540 (33.3)	179 (34.7)	361 (33.0)	
Missing	189 (1.1)	87 (1.0)	102 (1.2)		13 (0.8)	3 (0.6)	10 (0.9)	
CirCom score at HCC								
0	NA	NA	NA	NA	5 (0.3)	2 (0.4)	3 (0.3)	.06
1 + 0	NA	NA	NA		371 (22.9)	126 (24.3)	245 (22.2)	
1 + 1	NA	NA	NA		409 (25.2)	132 (25.4)	277 (25.1)	
3 + 0	NA	NA	NA		59 (3.6)	10 (1.9)	49 (4.0)	
3 + 1	NA	NA	NA		557 (34.3)	163 (31.4)	394 (35.7)	
5 + 0	NA	NA	NA		33 (2.0)	16 (3.1)	17 (1.5)	
5 + 1	NA	NA	NA		108 (6.7)	44 (8.5)	64 (5.8)	
Missing	NA	NA	NA		80 (4.9)	26 (5.0)	54 (4.9)	
Time since HCV cure at HCC diagnosis, y								
1 to <2	NA	NA	NA	NA	445 (27.4)	82 (15.8)	363 (32.9)	<.001
2 to <3	NA	NA	NA		377 (23.2)	112 (21.6)	265 (24.0)	
3 to <4	NA	NA	NA		310 (19.1)	101 (19.5)	209 (19.0)	
4 to <5	NA	NA	NA		227 (14.0)	101 (19.5)	126 (11.4)	
5 to <6	NA	NA	NA		135 (8.3)	55 (10.6)	80 (7.3)	
6 to <7	NA	NA	NA		98 (6.0)	49 (9.4)	49 (4.4)	
≥7	NA	NA	NA		30 (1.8)	19 (3.7)	11 (1.0)	
First HCC treatment								
Resection	NA	NA	NA	NA	74 (4.6)	27 (5.2)	47 (4.3)	.01
Ablation	NA	NA	NA		251 (15.5)	60 (11.6)	191 (17.3)	
Embolization	NA	NA	NA		724 (44.6)	204 (39.3)	520 (47.1)	
Radiation	NA	NA	NA		69 (4.3)	23 (4.4)	46 (4.2)	
Systemic or supportive	NA	NA	NA		504 (31.1)	205 (39.5)	299 (27.1)	

Abbreviations: CirCom, cirrhosis-specific comorbidity; HCC, hepatocellular carcinoma; HCV, hepatitis C virus; NA, not applicable.

^a^ Characteristics at baseline.

^b^ Characteristics at diagnosis.

^c^ *P* values for χ^2^ tests (categorical variables) and Wilcoxon rank-sum test (age) comparing individuals up to date with screening for less than 50% vs 50% or greater of screening eligible follow-up time.

^d^ *P* values for χ^2^ tests (categorical variables) and Wilcoxon rank-sum test (age) comparing individuals up to date with screening for less than 50% vs 50% or greater of the 2-year period preceding HCC diagnosis.

^e^ Other includes individuals who identified as any other race or ethnicity not otherwise specified or had unknown race and ethnicity.

### HCC Screening and Incidence

**Figure 1.**
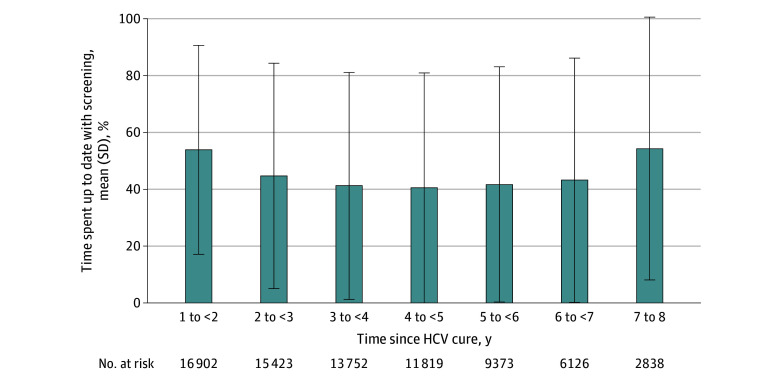
Mean Percentage of Time Eligible Participants Remained Up to Date With Hepatocellular Carcinoma Screening by Year Since Hepatitis C Virus (HCV) Cure Error bars denote SDs.

In the total sample, the mean (SD) percentage of time up to date with screening after HCV cure declined from 53.9% (36.8%) in year 1 to 40.5% (40.5%) in year 4, then increased to a maximum of 64.2% (48.0%) among individuals who remained screening eligible in year 8 (Figure 1). Individuals who were younger at the time of HCV cure spent, on average, less time up to date with screening (eTable 3 in Supplement 1). The annual cumulative incidence of HCC among individuals who remained eligible for screening after HCV cure gradually declined from 2.4% (409 of 16 902 individuals) during the first year of follow-up to 1.0% (27 of 2833 individuals) during year 8 (Table 2). A total of 270 individuals developed HCC 5 or more years after HCV cure, and among them, 53 deaths were observed.

**Table 2.** Annual Cumulative Incidence of HCC Among Those Who Remained Eligible for Screening by Year Since HCV Cure^a^

Outcome	Incidence by time since HCV cure, No.						
	1 to <2 y	2 to <3 y	3 to <4 y	4 to <5 y	5 to <6 y	6 to <7 y	7-8 y
No. at risk of HCC at year start	16 902	15 423	13 752	11 819	9365	6115	2833
HCC	409	352	287	206	123	90	27
Death	664	602	563	478	324	131	23
Child-Turcotte-Pugh class C cirrhosis	60	42	40	30	19	8	6
Metastatic cancer	162	147	123	116	59	27	10
End of follow-up	184	528	920	1624	2725	3026	2484
Annual cumulative incidence of HCC, mean (95% CI), %^a^^,^^b^	2.42 (2.19-2.65)	2.28 (2.05-2.52)	2.09 (1.85-2.33)	1.74 (1.51-1.98)	1.31 (1.08-1.54)	1.47 (1.17-1.77)	0.95 (0.60-1.31)

Abbreviations: HCC, hepatocellular carcinoma; HCV, hepatitis C virus.

^a^ Annual cumulative incidence of HCC refers to the cumulative incidence over each 1-year period expressed per 100 patient-years of follow-up.

^b^ Calculated using the normal approximation of the binomial distribution.

### Association of HCC Screening With Mortality Among Those Who Developed HCC

**Figure 2.**
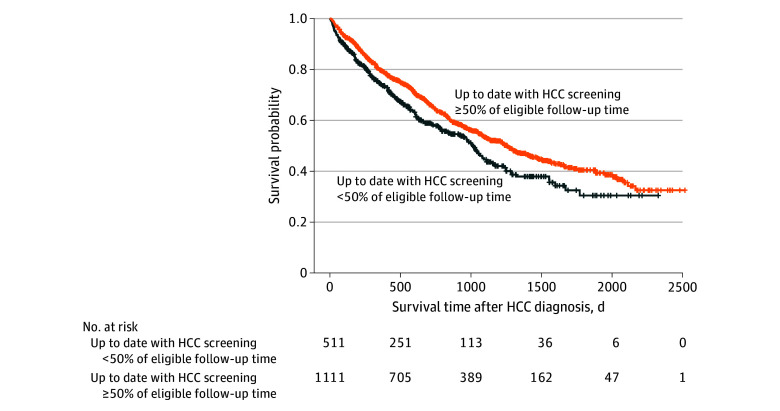
Overall Survival After Hepatocellular Carcinoma (HCC) Diagnosis Among Veterans With Hepatitis C Virus (HCV) Cirrhosis Who Achieved HCV Cure, by Percentage of Time Up to Date With Screening During the 4 Years Preceding HCC Diagnosis Kaplan-Meier survival curves are shown for individuals who were up to date with HCC screening for at least 50% of eligible follow-up vs those who were up to date with screening for less than 50% of eligible follow-up during the 4 years preceding HCC diagnosis. *P* = .002, log-rank test of equality over strata.

Of the 1622 individuals who developed HCC while eligible for screening and had complete covariable information, those who spent at least 50% of the time up to date with screening during the 4 years preceding HCC diagnosis had improved overall survival after HCC diagnosis (log-rank test of equality over strata *P* = .002) (Figure 2). In multivariable analysis, each 10% increase in the percentage of eligible follow-up spent up to date with screening during the 4 years preceding HCC diagnosis was associated with a 3.2% decrease in the hazard of death after HCC diagnosis (hazard ratio [HR], 0.97; 95% CI, 0.95-0.99) (Table 3 and full model results in eTable 4 in Supplement 1). There was a statistically significant interaction between the number of years since HCV cure and percentage of time up to date with screening (χ^2^ = 13.6; *P* = .03). Stratified by year since HCV cure, each 10% increase in the percentage of eligible follow-up spent up to date with screening was associated with a reduction in the hazard of death after HCC diagnosis of 8.0% in individuals who received a diagnosis of HCC 3 to less than 4 years after HCV cure (adjusted HR, 0.92; 95% CI, 0.87-0.98) and 13.0% in those who received a diagnosis 4 to less than 5 years after HCV cure (adjusted HR, 0.87; 95% CI, 0.79-0.95). There was a no association observed for those who received a diagnosis of HCC more than 5 years after HCV cure (Table 3). In sensitivity analyses, results were similar when evaluating screening during the 2 years preceding HCC diagnosis (eTable 5 in Supplement 1) and there was no statistically significant interaction between screening and age at HCC diagnosis (eTable 6 in Supplement 1). Accounting for lead time, overall survival after HCC diagnosis remained higher in those up to date with screening for 50% or more of eligible follow-up (log-rank test of equality over strata *P* = .04) (eFigure in Supplement 1). In adjusted survival analysis, each 10% increase in time up to date with screening was associated with a 2.0% decrease in the hazard of death after HCC diagnosis (adjusted HR, 0.98; 95% CI, 0.96-1.00) (eTable 7 in Supplement 1).

**Table 3.** Overall Survival After HCC Diagnosis Among Veterans With HCV-Associated Cirrhosis Who Achieved HCV Cure and Developed Incident HCC

Overall survival	Participants, No.	Person-y of follow-up, No.	Deaths, No.	Mortality per 100 person-y	Death, HR (95% CI)	
					Unadjusted	Adjusted^a^
By percentage of eligible time spent up to date with screening up to 4 y preceding HCC diagnosis^b^	1622	3350.8	731	21.8	0.97 (0.95-0.99)	0.97 (0.94-0.99)
By interaction between percentage of time spent up to date with screening and time since HCV cure^a^^,^^b^						
1 to <2 y	445	1215.4	254	20.9	NA	0.98 (0.95-1.01)
2 to <3 y	377	901.8	203	22.5	NA	0.98 (0.93-1.02)
3 to <4 y	310	599.0	155	25.9	NA	0.92 (0.87-0.98)
4 to <5 y	227	378.2	66	17.5	NA	0.87 (0.79-0.95)
5 to <6 y	135	150.0	34	22.7	NA	1.10 (0.96-1.26)
6 to <7 y	98	91.0	17	18.7	NA	0.98 (0.42-2.30)
≥7 y	30	15.5	2	12.9	NA	1.06 (0.90-1.26)

Abbreviations: HCC, hepatocellular carcinoma; HCV, hepatitis C virus; NA, not applicable.

^a^ Model adjusted for age at HCC diagnosis, sex, race and ethnicity, cause of cirrhosis, tobacco use, years since HCV cure, Child-Turcotte-Pugh class at HCC diagnosis, and cirrhosis-specific comorbidity score at HCC diagnosis.

^b^ Per 10% increase in time participants were up to date with screening.

In all, 864 of 1622 individuals with HCC were consistently undergoing screening for at least 50% of the time up to 4 years preceding HCC diagnosis. Additional time up to date with screening was not associated with overall survival after HCC diagnosis in this group (HR, 1.02; 95% CI, 0.94-1.11). Among those not consistently undergoing screening, each 10% increase in the percentage of time up to date with screening was associated with a 7.0% reduction in the hazard of death after HCC diagnosis (HR, 0.93; 95% CI, 0.88-0.99).

### Association of Screening With HCC Stage and Curative Treatment Receipt

Greater time spent up to date with screening during the 4 years preceding HCC diagnosis was associated with an increased likelihood of diagnosis with early-stage (AJCC stage I) HCC and receipt of potentially curative treatment (resection or ablation). For every 10% increase in percentage of time spent up to date with screening, individuals with HCC were 10.1% (95% CI, 6.3%-14.0%) more likely to receive a diagnosis of early-stage disease and 6.8% (95% CI, 2.8%-11.0%) more likely to receive curative treatment.

## Discussion

In this large, well-characterized cohort study of older individuals with HCV-associated cirrhosis, we observed a protective association of screening for HCC with overall survival after HCC diagnosis. Screening is thought to improve survival through detection of early-stage cancer amenable to curative therapy, and we observed that greater time spent up to date with screening was associated with an increased likelihood of early-stage HCC diagnosis and curative treatment receipt. Lack of recognition of cirrhosis contributes to low HCC screening rates,^11,12^ and these findings support efforts to identify cirrhosis among persons with cured HCV and engage appropriate patients in screening. Screening should be discontinued in patients with limited expected survival due to comorbid illness or CTP class C cirrhosis without liver transplant eligibility.

Few studies examining the outcomes of HCC screening distinguish individuals who have achieved HCV cure from those with chronic HCV, and individuals with treated HCV make up less than 10% of the study population in all but 1 study that makes this distinction.^10^ One evaluation of HCC screening and survival^32^ was performed in a research cohort of individuals with HCV-associated cirrhosis receiving liver imaging every 6 months. A total of 216 individuals developed HCC, but only 42 (19.9%) had achieved HCV cure at least 12 months before HCC diagnosis. Those who received a diagnosis of HCC more than 7 months from their last imaging study were characterized as nonadherent and experienced poorer overall survival after HCC diagnosis after adjusting for lead time.^32^ Our findings in this large cohort of individuals who achieved HCV cure, therefore, add substantially to the evidence evaluating survival outcomes associated with HCC screening in this population.

We also observed an association of percentage of time up to date with screening and improved survival after HCC diagnosis among individuals who were not consistently undergoing screening during eligible follow-up. Although rates of HCC screening in the VA health care system are higher than those in the private sector,^11,12,33^ only one-half of our cohort consistently received HCC screening. Thus, our findings are relevant to health care for nonveterans, who typically experience lower rates of HCC screening, and support interventions to improve screening engagement such as patient outreach.^34,35^

The positive association of screening with overall survival was only observed in individuals who developed HCC between 3 to less than 5 years after HCV cure. As the risk of HCC declines with time after HCV cure,^8^ individuals who develop HCC later may have a different risk profile. It is also possible that our study lacked statistical power to detect associations in these strata; only 270 individuals developed HCC 5 or more years after HCV cure and among them, only 53 deaths were observed. Longer duration of follow-up is needed to evaluate the association of screening with survival more than 5 years after HCV cure and whether discontinuation of screening should be recommended after an adequate interval.

### Limitations

Our study has several limitations, most notably its retrospective, observational nature. Despite efforts to address factors that would influence both participation in screening and overall survival at the analysis stage, residual confounding may be present. Individuals who received a diagnosis of HCC had high rates of comorbid disease (46.6% had a CirCom score of 3 + 0 or higher) and less than 5% underwent surgical resection, which are conditions that may have attenuated the observed survival benefit of screening. Female individuals are underrepresented in the VA population, and important sex differences may not be observed in this cohort. Although the use of semiannual α-fetoprotein measurement alongside liver imaging is currently recommended for HCC screening, this was not always the case, so for the sake of consistency over the study period, we evaluated imaging studies alone as representative of HCC screening status.

All evaluations of cancer screening and survival must consider potential biases influencing an observed survival benefit. Most observational studies of HCC screening have compared survival among persons who received a diagnosis of HCC on a screening examination with survival among those who received a diagnosis with symptoms.^10^ We instead measured the degree to which an individual received screening over the duration of time that they remained eligible, which is a more holistic measurement and does not rely on a single image in time. This approach protects, to a degree, against lead-time bias, which was further assessed by accounting for estimated lead time.

## Conclusions

In this cohort study of older adults with cirrhosis and cured HCV, HCC screening was associated with a survival benefit. As the population most impacted by HCV ages, the role of HCC screening after HCV cure requires ongoing evaluation to balance its potential harms and benefits. Our findings suggest that individuals with cirrhosis should be maintained in HCC screening after HCV cure.

## Notes

Supplement 1. **eTable 1.** Definition of CirCom Categories Based on Comorbidities Present **eTable 2.** Identification of Surgical and Interventional HCC Treatment Received **eTable 3.** Distribution of Percent of Time Up-to-Date With Screening During Eligible Follow-Up by Age at Index Date (HCV Cure) **eTable 4.** Overall Survival After HCC Diagnosis Among Veterans With HCV-Associated Cirrhosis Who Have Achieved HCV Cure and Develop Incident HCC (Full Results) **eTable 5.** Sensitivity Analysis Evaluating Percent of Time Up-to-Date With Screening During 2 Years Before HCC Diagnosis **eTable 6.** Proportional Hazard Regression Modeling Overall Survival on Percent of Eligible Follow-Up Up-to-Date With Screening During the 4 Years Preceding HCC Diagnosis Assessing for Interaction Between Age at HCC Diagnosis **eFigure.** Kaplan-Meier Survival Curve Corrected for Median Lead-Time Estimated for Annual Screening and Stratified by Percent of Time Up-to-Date With Screening During the 4 Years Preceding HCC Diagnosis **eTable 7.** Sensitivity Analysis Accounting for Median Lead-Time Estimated for Annual Screening
eReferences

Supplement 2.
Data Sharing Statement
