# Supplementary material for: Screening for Hepatocellular Carcinoma and Survival in Patients With Cirrhosis After Hepatitis C Virus Cure
Source: JAMA Netw Open. 2024 Jul 10;7(7):e2420963. doi: 10.1001/jamanetworkopen.2024.20963 (PMC11238019; doi:10.1001/jamanetworkopen.2024.20963)
Supplement: Supplement 1. — eTable 1. Definition of CirCom Categories Based on Comorbidities Present eTable 2. Identification of Surgical and Interventional HCC Treatment Received eTable 3. Distribution of Percent of Time Up-to-Date With Screening During Eligible Follow-Up by Age at Index Date (HCV Cure) eTable 4. Overall Survival After HCC Diagnosis Among Veterans With HCV-Associated Cirrhosis Who Have Achieved HCV Cure and Develop Incident HCC (Full Results) eTable 5. Sensitivity Analysis Evaluating Percent of Time Up-to-Date With Screening During 2 Years Before HCC Diagnosis eTable 6. Proportional Hazard Regression Modeling Overall Survival on Percent of Eligible Follow-Up Up-to-Date With Screening During the 4 Years Preceding HCC Diagnosis Assessing for Interaction Between Age at HCC Diagnosis eFigure. Kaplan-Meier Survival Curve Corrected for Median Lead-Time Estimated for Annual Screening and Stratified by Percent of Time Up-to-Date With Screening During the 4 Years Preceding HCC Diagnosis eTable 7. Sensitivity Analysis Accounting for Median Lead-Time Estimated for Annual Screening eReferences [file jamanetwopen-e2420963-s001.pdf]

Supplemental Online Content

Mezzacappa C, Kim NK, Vutien P, Kaplan DE, Ioannou GN, Taddei TH. Screening for hepatocellular carcinoma and survival in patients with cirrhosis after hepatitis C virus cure. *JAMA Netw. Open*. 2024;7(7):e2420963. doi:10.1001/jamanetworkopen.2024.20963

eTable 1. Definition of CirCom Categories Based on Comorbidities Present

eTable 2. Identification of Surgical and Interventional HCC Treatment Received

eTable 3. Distribution of Percent of Time Up-to-Date With Screening During Eligible Follow-Up by Age at Index Date (HCV Cure)

eTable 4. Overall Survival After HCC Diagnosis Among Veterans With HCV-Associated Cirrhosis Who Have Achieved HCV Cure and Develop Incident HCC (Full Results)

eTable 5. Sensitivity Analysis Evaluating Percent of Time Up-to-Date With Screening During 2 Years Before HCC Diagnosis

eTable 6. Proportional Hazard Regression Modeling Overall Survival on Percent of Eligible Follow-Up Up-to-Date With Screening During the 4 Years Preceding HCC Diagnosis Assessing for Interaction Between Age at HCC Diagnosis

eFigure. Kaplan-Meier Survival Curve Corrected For Median Lead-Time Estimated for Annual Screening and Stratified by Percent of Time Up-to-Date With Screening During the 4 Years Preceding HCC Diagnosis

eTable 7. Sensitivity Analysis Accounting for Median Lead-Time Estimated for Annual Screening

eReferences

This supplemental material has been provided by the authors to give readers additional information about their work.

© 2024 Mezzacappa C et al. *JAMA Network Open.*

eTable 1. Definition of CirCom Categories Based on Comorbidities Present

CirCom Score[1]	Definition
0	None of the following comorbidities: chronic obstructive pulmonary disease, acute myocardial infarction, peripheral arterial disease, epilepsy, substance use disorder other than alcohol use disorder, heart failure, cancer, or chronic kidney disease.
1+0	One of the above listed comorbidities.
1+1	More than one of the above listed comorbidities.
3+0	Active myocardial infarction, active non-metastatic or hematologic cancer, inactive metastatic cancer, or chronic kidney disease and no other above listed comorbidities.
3+1	Active myocardial infarction, active non-metastatic or hematologic cancer, inactive metastatic cancer, or chronic kidney disease and at least one of the above listed comorbidities.
5+0	Active metastatic cancer and no other above listed comorbidities.
5+1	Active metastatic cancer and at least one of the above listed comorbidities.

eTable 2. Identification of Surgical and Interventional HCC Treatment Received

Treatment	Data source	Data items
Resection	Inpatient procedure tables	CPT code 47100 (wedge resection)<br>CPT code 4712x (hepatectomy)<br>CPT code 47130 (hepatectomy)
Embolization<br>Ablation	Radiology tables	Text-based Structured Query Language queries (eg, “embolization,” “ablation”)

For additional details, see Supplemental Methods published by Serper et al.[2]

eTable 3. Distribution of Percent of Time Up-to-Date With Screening During Eligible Follow-Up by Age at Index Date (HCV Cure)

**Total sample with eradicated HCV and cirrhosis**		
**Age at index date**	**Median (IQR)**	**Mean (S.D.)**
< 60 years (n=3,741)	43.9% (21.0% - 75.4%)	47.7% (31.9%)
60 – <65 years (n=6,114)	51.7% (24.3% - 82.3%)	52.4% (32.8%)
65 – <70 years (n=5,176)	55.1% (26.1% - 83.9%)	54.3% (32.8%)
70 – <75 years (n=1,481)	57.4% (27.9% - 85.1%)	55.5% (33.1%)
≥ 75 years (n=390)	58.0% (28.2% - 86.4%)	56.5% (33.2%)
**Individuals who developed HCC**		
< 60 years (n=281)	77.9% (42.7% - 100.0%)	69.1% (31.8%)
60 – <65 years (n=577)	82.5% (48.0% - 100.0%)	72.1% (32.0%)
65 – <70 years (n=480)	90.8% (56.8% - 100.0%)	75.1% (30.4%)
70 – <75 years (n=128)	96.2% (61.4% - 100.0%)	77.8% (29.7%)
≥ 75 years (n=28)	98.6% (55.2% - 100.0%)	77.8% (30.0%)

© 2024 Mezzacappa C et al. *JAMA Network Open.*

eTable 4. Overall Survival After HCC Diagnosis Among Veterans With HCV-Associated Cirrhosis Who Have Achieved HCV Cure and Develop Incident HCC (Full Results)

**Predictor**	**N**	**Person-years of follow-up**	**Deaths**	**Mortality per 100 person-years**	**Adjusted[a] HR death (95% CI)**
Percent of time up to date with screening (per 10% increase)	1622	3350.8	731	21.8	0.97 (0.95-0.99)
Age at diagnosis of HCC (per 5-year increase)	1622	3350.8	731	21.8	1.02 (0.94-1.11)
Sex					
Male	1589	3284.2	713	21.7	0.69 (0.43-1.11)
Female	33	66.7	18	27.0	Ref.
Race/ethnicity					
White	881	1794.5	420	23.4	Ref.
Black	457	967.5	191	19.7	0.77 (0.65-0.92)
Hispanic	132	272.3	61	22.4	0.85 (0.65-1.12)
Asian/Pac. Islander	22	51.1	10	19.6	0.89 (0.47-1.67)
Nat. American	20	51.2	10	19.5	0.86 (0.46-1.62)
Other	110	214.3	39	18.2	0.81 (0.58-1.14)
Etiology of cirrhosis					
HCV alone	856	1802.2	370	20.5	Ref.
HCV + alcohol	766	1548.6	361	23.3	1.05 (0.90-1.22)
Tobacco use					
Never	462	955.6	213	22.3	Ref.
Former	607	1304.9	263	20.2	1.08 (0.83-1.20)
Current	540	1062.3	252	23.7	1.00 (0.90-1.28)
Years since HCV cure					
1-2					
2-3	445	1215.4	254	20.9	Ref.
3-4	377	901.8	203	22.5	0.99 (0.82-1.20)
4-5	310	599.0	155	25.9	1.06 (0.87-1.31)
5-6	227	378.2	66	17.5	0.65 (0.49-0.86)
6-7	135	150.0	34	22.7	0.81 (0.56-1.19)
7+	98	91.0	17	18.7	0.65 (0.39-1.07)
	30	15.5	2	12.9	0.35 (0.09-1.40)
CTP class at HCC					
A	1430	3055.7	611	20.0	Ref.
B	192	295.1	120	40.7	1.93 (1.57-2.36)
CirCom at HCC diagnosis					
0	5	11.6	3	25.9	1.19 (0.37-3.81)
1+0	319	664.0	128	19.3	Ref.
1+1	372	851.9	150	17.6	0.89 (0.71-1.11)
3+0	58	129.5	25	19.3	1.05 (0.68-1.62)
3+1	502	1158.3	205	17.7	0.97 (0.79-1.20)
5+0	33	26.4	25	94.6	4.95 (3.22-7.63)
5+1	107	106.7	77	72.1	3.44 (2.58-4.59)

© 2024 Mezzacappa C et al. *JAMA Network Open.*

eTable 5. Sensitivity Analysis Evaluating Percent of Time Up-to-Date With Screening During 2 Years Before HCC Diagnosis
Overall survival after HCC diagnosis among Veterans with HCV-associated cirrhosis who have achieved HCV cure and develop incident HCC

**Predictor**	**N**	**Person-years of follow-up**	**Deaths**	**Mortality per 100 person-years**	**Unadjusted HR death (95% CI)**	**Adjusted[a] HR death (95% CI)**
Percent of time up to date with screening during up to 2 years preceding HCC diagnosis (per 10% increase)	1622	3350.8	731	21.8	0.97 (0.95-0.99)	0.97 (0.94-0.99)
**Overall survival by interaction between percent of time up to date with screening and years since HCV cure[a]**						
	**N**	**Person-years of follow-up**	**Deaths**	**Mortality per 100 person-years**	**Adjusted[a] HR death for 10% increase in eligible time up to date with screening (95% CI)**	
**Years since HCV cure**						
1-2	445	1215.4	254	20.9	0.97 (0.93-1.00)	
2-3	377	901.8	203	22.5	0.97 (0.92-1.01)	
3-4	310	599.0	155	25.9	0.96 (0.92-0.98)	
4-5	227	378.2	66	17.5	0.90 (0.84-0.97)	
5-6	135	150.0	34	22.7	1.10 (0.98-1.25)	
6-7	98	91.0	17	18.7	0.99 (0.87-1.14)	
7+	30	15.5	2	12.9	0.75 (0.52-1.08)	

[a]Model adjusted for age at HCC diagnosis, sex, race/ethnicity, etiology of cirrhosis, tobacco use, years since HCV cure, CTP class at HCC diagnosis, CirCom at HCC diagnosis.

eTable 6. Proportional Hazard Regression Modeling Overall Survival on Percent of Eligible Follow-Up Up-to-Date With Screening During the 4 Years Preceding HCC Diagnosis Assessing for Interaction Between Age at HCC Diagnosis[a]

	Age at HCC <70		Age at HCC ≥70	
	HR death	95% CI	HR death	95% CI
Percent of eligible time up to date with screening in 2 years preceding HCC diagnosis (per 10% increase)	0.98	0.96 – 1.00	0.95	0.90 – 1.00
[a]p-value interaction term between percent of time up to date with screening and age = 0.26				
	Age at HCC <75		Age at HCC ≥75	
	HR death	95% CI	HR death	95% CI
Percent of eligible time up to date with screening in 2 years preceding HCC diagnosis (per 10% increase)	0.97	0.95 – 0.99	0.96	0.86 – 1.08
[a]p-value interaction term between percent of time up to date with screening and age = 0.84				

Model adjusted for number of years since HCV cure, CTP class at HCC diagnosis, CirCom at HCC diagnosis, etiology of cirrhosis, sex, race and ethnicity, tobacco use.

© 2024 Mezzacappa C et al. *JAMA Network Open.*

eFigure . Kaplan-Meier Survival Curve Corrected For Median Lead-Time Estimated for Annual Screening and Stratified by Percent of Time Up-to-Date With Screening During the 4 Years Preceding HCC Diagnosis

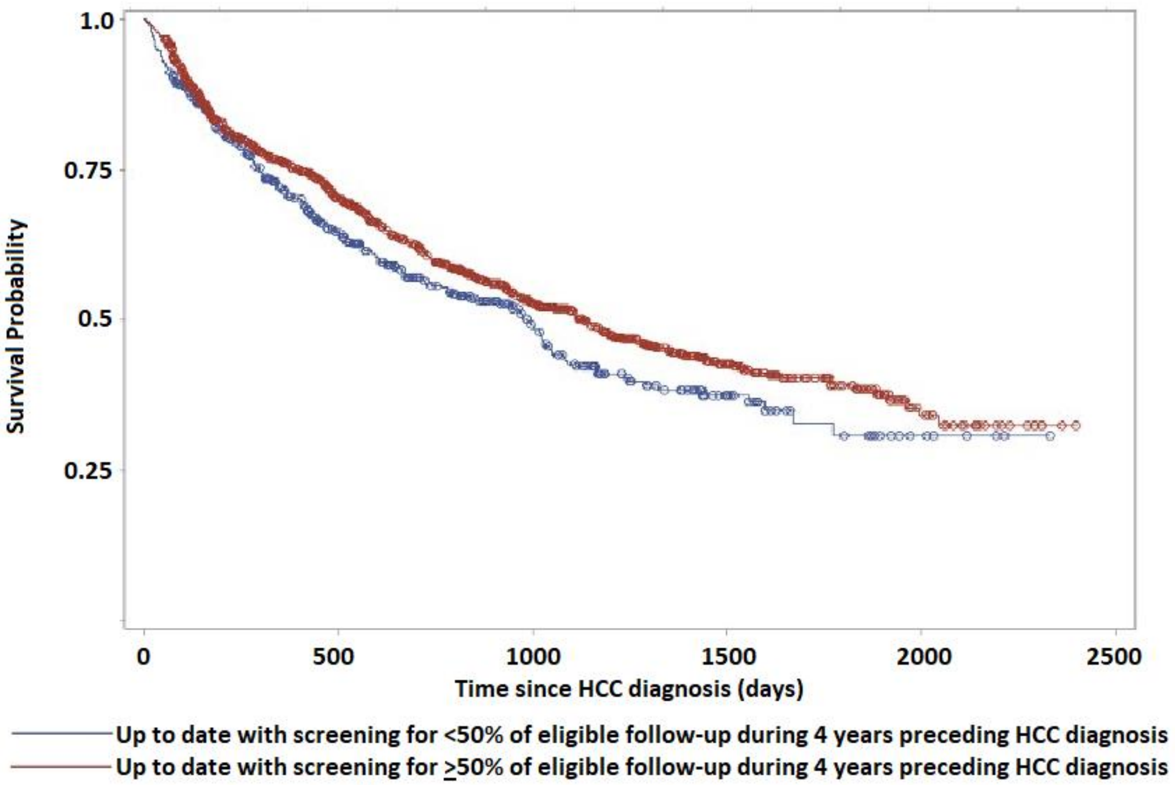

eTable 7. Sensitivity Analysis Accounting for Median Lead-Time Estimated for Annual Screening Overall survival after HCC diagnosis among Veterans with HCV-associated cirrhosis who have achieved HCV cure and develop incident HCC

**Predictor**	**N**	**Person-years of follow-up**	**Deaths**	**Mortality per 100 person-years**	**Adjusted[a] HR death (95% CI)**
Percent of eligible time up to date with screening during up to 4 years preceding HCC diagnosis (per 10% increase)	1622	3350.8	731	21.8	0.98 (0.96 – 1.00)

[a]Model adjusted for age at HCC diagnosis, sex, race/ethnicity, etiology of cirrhosis, tobacco use, years since HCV eradication, CTP class at HCC diagnosis, CirCom at HCC diagnosis.

© 2024 Mezzacappa C et al. *JAMA Network Open.*

eReferences

1. Jepsen P, Vilstrup H, Lash TL. Development and validation of a comorbidity scoring system for patients with cirrhosis. *Gastroenterology*. Jan 2014;146(1):147-56; quiz e15-6. doi:10.1053/j.gastro.2013.09.019

2. Serper M, Taddei TH, Mehta R, et al. Association of Provider Specialty and Multidisciplinary Care With Hepatocellular Carcinoma Treatment and Mortality. *Gastroenterology*. Jun 2017;152(8):1954-1964. doi:10.1053/j.gastro.2017.02.040

© 2024 Mezzacappa C et al. *JAMA Network Open.*
