# Supplementary material for: Screening for Hepatocellular Carcinoma and Survival in Patients With Cirrhosis After Hepatitis C Virus Cure
Source: JAMA Netw Open. 2024 Jul 10;7(7):e2420963. doi: 10.1001/jamanetworkopen.2024.20963 (PMC11238019; doi:10.1001/jamanetworkopen.2024.20963)
Supplement: Supplement 2. — Data Sharing Statement [file jamanetwopen-e2420963-s002.pdf]

Data Sharing Statement

Mezzacappa. Screening for Hepatocellular Carcinoma and Survival in Patients With Cirrhosis After Hepatitis C Virus Cure. *JAMA Netw Open*. Published July 10, 2024.
doi:10.1001/jamanetworkopen.2024.20963

Data
Data available: No

Additional Information
Explanation for why data not available: Due to US Department of Veterans Affairs (VA) regulations, data originating in VA-approved studies are the property of the VA and any data sharing requires approval from the VA and development of a Data Use Agreement (DUA). The corresponding or senior author can be contacted directly to request creation of a DUA, which is required to share identifiable human subjects data outside of the VA. For more information, please visit https://www.virec.research.va.gov.
